# Exploring how to widen the acceptability of public health interventions: a systematic review protocol

**DOI:** 10.1136/bmjopen-2024-088418

**Published:** 2024-11-09

**Authors:** Kaitlin Conway-Moore, Fiona Graham, Alison R McKinlay, Jack Birch, Emily Oliver, Clare Bambra, Michael P Kelly, Chris Bonell

**Affiliations:** 1 NIHR Policy Research Unit Behavioural and Social Sciences, Department of Public Health, Environments and Society, London School of Hygiene and Tropical Medicine, London, UK; 2 NIHR Policy Research Unit Behavioural and Social Sciences, Population Health Sciences Institute, Faculty of Medical Sciences, Newcastle University, Newcastle upon Tyne, UK; 3 NIHR Policy Research Unit Behavioural and Social Sciences, Centre for Behaviour Change, Department of Clinical, Educationaland Health Psychology, University College London, London, UK; 4 Department of Public Health and Primary Care, University of Cambridge, Cambridge, UK

**Keywords:** Health policy, Public health, Social medicine

## Abstract

**Abstract:**

**Introduction:**

Health interventions that require significant change to individual lifestyles or social norms can pose a challenge for widespread public acceptability and uptake. At the same time, over the last two decades, there has been increasing attention paid to the rise of populist movements globally, defined by ‘the people’ pushing against ‘an elite’ viewed as depriving the people of their sovereignty. To understand potential overlap in these two areas, this study aims to synthesise existing international evidence on linkages between populist attitudes and reduced uptake, acceptability, adherence and/or effectiveness of public health interventions. The goal of this work is to create a conceptual framework that can be used to inform policy strategies aimed at widening the impact of public health interventions.

**Methods and analysis:**

A systematic review will be performed via searches across databases and websites relevant to public health and social science research, informed by preliminary searches on the topic. There will be no language restrictions, but included studies will be limited to those produced since 2008, the year of the global financial crisis, from which most current literature on populism dates. Risk of bias will be assessed using validated tools according to study design. Due to expected heterogeneity across included studies, this will be a systematic review without meta-analysis. Findings will be synthesised narratively, and the strength of the evidence will be assessed using the Grading of Recommendations Assessment, Development and Evaluation approach. The review will be reported according to the Systematic Reviews without Meta-Analysis reporting guidelines.

**Ethics and dissemination:**

Ethical review is not required for this study. Public dissemination will be informed via consultation with our Patient and Public Involvement and Engagement Strategy Group, along with reporting via peer-reviewed publication, relevant international conferences, a policy brief and a workshop with public health and communications experts.

**PROSPERO registration number:**

CRD42024513124.

**STRENGTHS AND LIMITATIONS OF THIS STUDY**

- This systematic review on the impact of populist attitudes on the acceptability and uptake of public health interventions is informed by preliminary searches on the same topic and uses a peer-reviewed search strategy.
- The review adheres to the Systematic Reviews without Meta-Analysis reporting guidelines.
- Findings from this review can be used to better inform future public health interventions to increase both individual and population-level acceptability and uptake.
- A limitation of this review lies in the fact that the expected heterogeneity of included studies will prohibit meta-analysis.

## Introduction

 The widespread acceptability of public health interventions is key to their uptake and success. However, achieving broad-based public support can be challenging when interventions require significant change to individual lifestyles or social norms. This was made evident in a 2013 systematic review on the public acceptability of government interventions aimed at changing health-related behaviours such as tobacco and alcohol consumption, diet and exercise.^1^ In this review, it was found that people tended to be most receptive to interventions that were the least intrusive to their lives or targeted towards the behaviour of others, even though these types of interventions also tended to be the least effective.^1^

One of the most salient examples of the potentially negative impact of a lack of acceptability and uptake of public health interventions occurred in 2019, when the WHO declared vaccine hesitancy among the top ten threats to global health.^2^ Along with vaccine hesitancy, however, there have recently been instances of public protest and backlash against other government-led health interventions in countries around the world. Among these are climate change mitigation strategies, sexual and reproductive healthcare provision, and non-pharmaceutical-based infection control measures, with negative reactions having the potential to contribute to further uncertainties about and scepticism towards, public health interventions globally.^3^,^7^

One potential driver of this phenomenon could be connected to the rise of populist politics and attitudes over the last two decades. Responding and adding to widespread dissatisfaction with, and alienation from, existing government structures, populist politics are generally constructed by those making such appeals in terms of ‘the people’ standing in opposition to an ‘elite’ who are viewed as depriving the people of their sovereignty or pushing for unwelcome social change.^8 9^ While who is said to make up ‘the people’ and who ‘the elite’ varies and can change over time and across the political spectrum, this matters less than the clear distinction of ‘us’ versus ‘them’.^10^ In left-wing populism, the distinction is generally binary, with people economically in the middle and bottom of society presented as pushing against those at the top. In right-wing populism, on the other hand, the distinction is more often triadic, with ‘the people’ presented as pushing upward against ‘the elite’ and downward against a specific ‘other’ or ‘out group’, the latter of which often includes immigrants, ethnic/religious/linguistic minorities, or members of the LGBTQ+ community.^11 12^

While much of the current discourse about populist movements and attitudes has centred on their impact on social cohesion and illiberalism, there may be important implications for public health that require examination. In particular, evidence suggests that support for conspiracy theories may have an important role to play in opposition to public health interventions and one that can be connected directly to populist attitudes through a focus on the alleged wrongdoings of existing institutions, elites and authorities that lie at the root of many such theories.^13^ It has been argued that this focus is particularly relevant when the scientific data being presented in support of public health interventions threaten an individual’s worldview or ideologies or when it has an impact on public policy, as promoting conspiracy theories provides room for rejecting the information being provided by experts.

Interestingly, when looking at the core drivers of scepticism concerning issues such as climate change, vaccination or COVID-19 restrictions, existing evidence suggests that they are often different.^13^ For example, while climate change scepticism tends to be rooted in conservative or neo-liberal political values, and especially support for an economy free from the kinds of regulations that commonly proposed environmental protection measures might involve, other factors have been found to be more powerful drivers of vaccine scepticism or hesitancy, such as religiosity, gender, ethnic minority status, educational attainment and a lack of trust in both government and scientists.^13^,^16^ When it comes to COVID-19 restrictions, worldview and group identity have been noted as major drivers of scepticism.^13^ More specifically, it has been argued that opposition to mandatory self-isolation, facemask requirements, and limitations to travel, leisure and social activities mainly came from those who generally oppose government intervention in the lives of citizens, limitations to personal freedom and resulting social change.^13 17 18^

To better understand the role that populist attitudes play in affecting the acceptability and uptake of different public health interventions, there is a need for a synthesis of empirical evidence, including both quantitative and qualitative research. To this end, the present protocol outlines a systematic review that seeks to answer the following research question: what existing international evidence is there for statistical associations or thematic linkages between populist attitudes and the reduced uptake, acceptability, adherence and/or effectiveness of public health interventions?

While a set of preliminary searches of published literature conducted by the authors of this protocol, along with media coverage on the topic, has identified interventions focused on vaccination, climate change, sexual and reproductive healthcare, non-pharmaceutical-based infection control, tobacco and alcohol consumption, diet and exercise as key areas of focus, this study will take a broader approach to include all potentially relevant public health interventions. The findings can then be harnessed into a conceptual framework that can be used to inform policy strategies aimed at overcoming resistance to public health interventions by those with populist attitudes, with the goal of improving health outcomes at both the individual and population level.

## Methods and analysis

### Research design overview

The review will follow the Systematic Reviews without Meta-Analysis (SWiM) reporting guidelines (see online supplemental appendix 1 for the SWiM reporting items).^19^ The protocol for this review has been registered with PROSPERO International Prospective Register of Systematic Review, registration number CRD42024513124.

### Inclusion criteria

#### Types of participants

Given our desire to ultimately identify evidence applicable to a UK policy context, studies will be included if participants are adults living in countries as defined by membership in the Organisation for Economic Co-operation and Development.

#### Types of interventions

To be included, studies will need to focus on a specific public health intervention, such as those addressing vaccination, disease screening, non-pharmaceutical infection controls, sexual/reproductive healthcare provision, increased access to healthcare, climate change mitigation, road safety, anti-pollution measures, water fluoridation, gun control, mental healthcare provision, improvements to diet and exercise, and those related to gambling, as well as tobacco and alcohol or other drug use.

#### Types of exposure or moderator

Included studies will have a focus on participant attitudes which can be construed as populist in the sense of being hostile to at least one of the following: (1) elites (eg, government, business, medical and other health professionals, mainstream media, science and the wealthy), (2) out-groups (eg, women, migrants, minoritised ethnic/racial/religious groups or gender/sexual minorities), (3) checks on popular sovereignty (eg, legal rights, personal freedoms and other government-imposed regulations) or (4) social change (eg, promotion of equality, diversity and inclusion, state intervention or market regulation).

#### Types of outcomes

The outcome(s) of interest are uptake of, acceptability of, adherence to and/or effectiveness of public health interventions. To be included, studies will need to report on the impact of populist attitudes on one or more of these outcome measures.

#### Types of study design

This study will include any report of empirical research that uses the following research design: (1) trials or other before and after (controlled and uncontrolled) evaluations of public health interventions examining whether populist attitudes moderate or otherwise affect intervention uptake, acceptability, adherence and/or effectiveness; (2) cross-sectional, longitudinal or discrete choice studies examining associations between populist attitudes and the uptake, acceptability, adherence and/or effectiveness of public health interventions; or (3) qualitative research (eg, interviews, focus groups, ethnographies) examining linkages between populist views and views on or behaviour/practices in relation to public health interventions.

### Search strategy

Our search strategy is informed by a preliminary set of searches we conducted on how populist attitudes relate to the uptake, acceptability, adherence or effectiveness of public health interventions, which was used to establish the existence of pertinent evidence on this topic and to inform search terms. Based on this preliminary set of searches, we will limit this systematic review to literature published since 2008, the year of the global financial crisis, from which the contemporary literature on populism largely dates. We will not limit our search by language or publication type.

In terms of bibliographic database searches, we will mainly use free-text terms as studies meeting our inclusions criteria are unlikely to be reliably indexed in databases with controlled vocabularies such as Medical Subject Headings. We will take the following concepts from our inclusion criteria to develop a search string linked by ‘AND’: populist attitude AND public health intervention AND outcome. For each concept, we will use free-text and controlled-vocabulary terms linked by ‘OR’. The following databases will searched, based on their relevance to medical, psychological, economic and social scientific research: CINAHL; Dissertation Abstracts; EconLit; EMBASE; Global Health; Global Index Medicus; International Bibliography of the Social Sciences; Ovid MEDLINE; PsycINFO; Scopus; Social Policy and Practice; Sociological Research Online; and Web of Science (including Science Citation Index Expanded, Social Sciences Citation Index, Arts & Humanities Citation Index and Emerging Sources Citation Index). An example of the final search strategy employed in Ovid MEDLINE and peer-reviewed by a librarian at the London School of Hygiene and Tropical Medicine can be found in online supplemental appendix 2.

We will also run searches on the following websites to retrieve relevant grey literature: Centers for Disease Control and Prevention; Community Research and Development Information Service; Drug and Alcohol Findings Effectiveness Bank; European Centre for Disease Prevention and Control; Google; Google Scholar; Intergovernmental Panel on Climate Change; International Planned Parenthood Federation; Marie Stopes International; The Campbell Library; Open Library; United Nations Environment Programme; and the World Health Organization.

Reference lists of all included studies will be hand-searched for additional studies that meet our inclusion criteria. Finally, we will contact subject experts for relevant ongoing or completed research and anything that may have been missed in our systematic searching outlined above.

### Data management and screening

Results of comprehensive searching will be downloaded into EPPI Reviewer 6^20^ and duplicates will be removed. Two reviewers will pilot the screening of successive batches of 50 titles and abstracts, meeting to discuss disagreements and calling on a third reviewer to settle disagreements where necessary. After refinements and once we achieve a batch-level agreement rate of 90%, each record will be screened by one reviewer for potential inclusion based on its title and abstract. Full texts will be obtained for references judged as meeting our inclusion criteria or where there is insufficient information from the title and abstract to decide. Screening of full texts will follow the same process outlined for title and abstract screening.

### Data extraction

Data extraction will be carried out by one reviewer using EPPI Reviewer 6^20^ with cross-checking conducted by a second reviewer. The following data will be extracted from each included record: basic study details (ie, first author, publication date, study location, timing and duration, individual participant characteristics); study methods (ie, design (including intervention type), sampling and sample size, allocation, blinding, control of confounding, data collection, attrition, analysis); outcome measures (ie, outcome type (eg, focus on uptake, acceptability, adherence or effectiveness of public health interventions), timing, reliability of measures, intra-class correlation coefficients, effect sizes); and relevant moderation analyses. If included studies are reported in languages that cannot be translated by the review team, a review author will complete the data extraction form in collaboration with a translator. In the case of missing data, we will contact the authors of the document in question and request the missing details. If authors cannot be traced or do not respond after a period of 2 months, we will record the information as missing in our extraction sheet and take this into account in our risk of bias assessment for that record.

### Study quality and risk of bias appraisal

Two reviewers will independently assess the quality of each study and then meet to compare their assessments. Disagreements will be resolved through discussion and, where necessary, via a third reviewer. Depending on the study design, different quality assessment and risk of bias tools will be employed. For randomised controlled trials, we will use the Cochrane Risk of Bias (RoB 2) tool, which assesses domains of bias in trial design, conduct and reporting.^21^ For non-randomised studies and quasi-experimental evaluations, we will use the Risk of Bias in Non-Randomized Studies - of Interventions tool.^22^ This tool evaluates studies based on seven domains of potential bias, including confounding, selection bias, intervention measurement, deviations from intended interventions, missing data, outcome measurement and reported results.^23^ For quantitative observational studies including cohort, cross-sectional and case-control designs, we will use the Cambridge Quality Checklists, which evaluate study methods used in determining correlates, risk factors and causal risk factors.^24^ Qualitative studies will be assessed using the EPPI Centre’s quality assessment evaluation tool.^25^

### Data analysis and synthesis

As this is a multi-methods review including both quantitative and qualitative research and based on the heterogeneity of quantitative evidence identified in our preliminary searches, this will be a systematic review without meta-analysis, with all findings synthesised narratively. Narrative synthesis will focus on how the uptake, acceptability, adherence and/or effectiveness of public health interventions appear to be affected by the holding of populist views among potential beneficiaries within each study. Where possible, we will explore the reasons for any differences found between those who hold and do not hold populist views in each study. As part of this synthesis, we will also reflect on the nature of the interventions on which the included studies report, as these may be different based on study setting/location. Results will be ordered by topic as appropriate, including vaccination; disease screening; infection control measures; sexual and/or reproductive health; increased access to healthcare; climate change; road safety; water fluoridation; gun control; mental health; diet and exercise; gambling; and tobacco, alcohol and other drug use, among others. Where appropriate, we will use meta-ethnographic methods to inform our narrative synthesis of qualitative evidence. The overarching aim of this narrative synthesis will be the development of a conceptual framework that can be used to inform policy strategies aimed at widening the impact of key public health interventions.

Characteristics of all the studies included in this review will be presented in a table that outlines the authorship, publication date, study design, setting, sample population and size, as well as type(s) of intervention, exposure(s)/moderator(s) and outcome(s). The strength of the evidence presented in our review will be assessed using the Grading of Recommendations Assessment, Development and Evaluation (GRADE) approach^26^ for quantitative studies, and the Confidence in the Evidence from Reviews of Qualitative Research framework^27^ for qualitative studies, which will be included in a table outlining our summary findings for each outcome of interest. GRADE assessments will examine the quality of evidence based on five domains (eg, risk of bias, inconsistency, indirectness, imprecision and publication bias) and will result in a given outcome being placed in one of four levels of certainty based on the presented body of evidence (eg, high, moderate, low or very low).^28^ Depending on the number of studies found for each outcome, we will also create funnel plots to assess whether there is evidence of publication bias.

### Patient and public involvement

As a project funded under the National Institute for Health and Care Research Policy Research Unit Behavioural and Social Sciences, this study has a designated Patient and Public Involvement and Engagement (PPIE) representative who will be regularly consulted to ensure their robust engagement with the research process, including the planning of future qualitative research that will complement the present study. To date, collaboration with our PPIE representative has included the production of a lay summary of the research project and critical revision of this protocol, with feedback incorporated in order to strengthen engagement with a wide audience. Following execution of the review, findings will also be presented to our PPIE Strategy Group to understand the extent to which the findings align with their own experiences, to produce a lay summary of the findings for broader public engagement with the research outputs and to identify public dissemination opportunities.

### Ethics and dissemination

The research involves no human participants and draws solely on evidence available in the public domain, so ethical approval is not required for this project. Dissemination of findings will include presentation to our PPIE Strategy Group, peer-reviewed publications in international journals, presentations at international public health and social science conferences, a policy brief and a workshop with public health and communications experts to relay findings and recommendations for improved uptake and acceptability of future public health interventions.

## supplementary material

10.1136/bmjopen-2024-088418 online supplemental file 1
