# Supplementary material for: Exploring how to widen the acceptability of public health interventions: a systematic review protocol
Source: BMJ Open. 2024 Nov 9;14(11):e088418. doi: 10.1136/bmjopen-2024-088418 (PMC11551972; doi:10.1136/bmjopen-2024-088418)
Supplement: online supplemental file 1 [file bmjopen-14-11-s001.docx]

# Appendix 1: Synthesis Without Meta-analysis (SWiM) reporting items^[[1]](#footnote-1)^

| **SWiM is intended to complement and be used as an extension to PRISMA** | | | |
| --- | --- | --- | --- |
| **SWiM reporting item** | **Item description** | **Page in manuscript where item is reported** | **Other*** |
| *Methods* | | | |
| **1** Grouping studies for synthesis | 1a) Provide a description of, and rationale for, the groups used in the synthesis (e.g., groupings of populations, interventions, outcomes, study design) |  |  |
|  | 1b) Detail and provide rationale for any changes made subsequent to the protocol in the groups used in the synthesis |  |  |
| **2** Describe the standardised metric and transformation methods used | Describe the standardised metric for each outcome. Explain why the metric(s) was chosen, and describe any methods used to transform the intervention effects, as reported in the study, to the standardised metric, citing any methodological guidance consulted |  |  |
| **3** Describe the synthesis methods | Describe and justify the methods used to synthesise the effects for each outcome when it was not possible to undertake a meta-analysis of effect estimates |  |  |
| **4** Criteria used to prioritise results for summary and synthesis | Where applicable, provide the criteria used, with supporting justification, to select the particular studies, or a particular study, for the main synthesis or to draw conclusions from the synthesis (e.g., based on study design, risk of bias assessments, directness in relation to the review question) |  |  |
| **SWiM reporting item** | **Item description** | **Page in manuscript where item is reported** | **Other*** |
| **5** Investigation of heterogeneity in reported effects | State the method(s) used to examine heterogeneity in reported effects when it was not possible to undertake a meta-analysis of effect estimates and its extensions to investigate heterogeneity |  |  |
| **6** Certainty of evidence | Describe the methods used to assess certainty of the synthesis findings |  |  |
| **7** Data presentation methods | Describe the graphical and tabular methods used to present the effects (e.g., tables, forest plots, harvest plots).  Specify key study characteristics (e.g., study design, risk of bias) used to order the studies, in the text and any tables or graphs, clearly referencing the studies included |  |  |
| *Results* | | | |
| **8** Reporting results | For each comparison and outcome, provide a description of the synthesised findings, and the certainty of the findings. Describe the result in language that is consistent with the question the synthesis addresses, and indicate which studies contribute to the synthesis |  |  |
| *Discussion* |  |  |  |
| **9** Limitations of the synthesis | Report the limitations of the synthesis methods used and/or the groupings used in the synthesis, and how these affect the conclusions that can be drawn in relation to the original review question |  |  |

PRISMA=Preferred Reporting Items for Systematic Reviews and Meta-Analyses.

*If the information is not provided in the systematic review, give details of where this information is available (e.g., protocol, other published papers (provide citation details), or website (provide the URL)).

# Appendix 2: Ovid MEDLINE search strategy^^[[2]](#footnote-2)^^

| **#** | **Query** | **Results from 15 Feb 2024** |
| --- | --- | --- |
| 1 | Populis*.ti,ab. | 3,760 |
| 2 | Illiberal*.ti,ab. | 378 |
| 3 | Anti-liberal*.ti,ab. | 36 |
| 4 | Antiliberal*.ti,ab. | 23 |
| 5 | Anti-right*.ti,ab. | 30 |
| 6 | Antiright*.ti,ab. | 1 |
| 7 | (Hostil* adj2 right*).ti,ab. | 15 |
| 8 | (Hostil* adj2 liberal*).ti,ab. | 6 |
| 9 | Anti-EDI*.ti,ab. | 70 |
| 10 | (Hostil* adj2 EDI*).ti,ab. | 7 |
| 11 | Anti-equalit*.ti,ab. | 14 |
| 12 | Antiequalit*.ti,ab. | 0 |
| 13 | (Hostil* adj2 equalit*).ti,ab. | 7 |
| 14 | Anti-divers*.ti,ab. | 12 |
| 15 | Antidivers*.ti,ab. | 3 |
| 16 | (Hostil* adj2 divers*).ti,ab. | 65 |
| 17 | Anti-inclu*.ti,ab. | 19 |
| 18 | Anti-intervent*.ti,ab. | 20 |
| 19 | (Hostil* adj2 intervent*).ti,ab. | 126 |
| 20 | Anti-regulat*.ti,ab. | 110 |
| 21 | Antiregulat*.ti,ab. | 41 |
| 22 | (Hostil* adj2 regulat*).ti,ab. | 83 |
| 23 | (Hostil* adj2 inclu*).ti,ab. | 653 |
| 24 | Racism/ | 32,224 |
| 25 | Racis*.ti,ab. | 42,933 |
| 26 | Scapegoating/ | 282 |
| 27 | Xenophobia/ | 1,384 |
| 28 | Sexism/ | 11,631 |
| 29 | Elitis*.ti,ab. | 1,725 |
| 30 | Sovereignty.ti,ab. | 6,425 |
| 31 | Nationalis*.ti,ab. | 9,001 |
| 32 | Nativis*.ti,ab. | 1,327 |
| 33 | Pro-nativis*.ti,ab. | 1 |
| 34 | Pronativis*.ti,ab. | 0 |
| 35 | Anti-immigra*.ti,ab. | 1,636 |
| 36 | (Hostil* adj2 immigra*).ti,ab. | 128 |
| 37 | Anti-migra*.ti,ab. | 5,022 |
| 38 | Antimigra*.ti,ab. | 4,184 |
| 39 | (Hostil* adj2 migra*).ti,ab. | 41 |
| 40 | Anti-minorit*.ti,ab. | 61 |
| 41 | Antiminorit*.ti,ab. | 22 |
| 42 | (Hostil* adj2 minorit*).ti,ab. | 33 |
| 43 | Anti-ethnic*.ti,ab. | 10 |
| 44 | Antiethnic*.ti,ab. | 1 |
| 45 | (Hostil* adj2 ethnic*).ti,ab. | 94 |
| 46 | (Hostil* adj2 raci*).ti,ab. | 228 |
| 47 | Homophobia/ | 7,550 |
| 48 | Homophobi*.ti,ab. | 9,221 |
| 49 | Transphobi*.ti,ab. | 1,707 |
| 50 | Anti-globali*.ti,ab. | 203 |
| 51 | Antiglobali*.ti,ab. | 69 |
| 52 | (Hostil* adj2 globali*).ti,ab. | 1 |
| 53 | (Hostil* adj2 wealth*).ti,ab. | 1 |
| 54 | Anti-cosmopolitan.ti,ab. | 6 |
| 55 | Anticosmopolitan.ti,ab. | 0 |
| 56 | Far-right.ti,ab. | 977 |
| 57 | Anti-establishment.ti,ab. | 161 |
| 58 | Antiestablishment.ti,ab. | 61 |
| 59 | (Hostil* adj2 establishment*).ti,ab. | 2 |
| 60 | (Trust* adj2 establishment*).ti,ab. | 326 |
| 61 | Brexit.ti,ab. | 3,836 |
| 62 | Anti-expert*.ti,ab. | 27 |
| 63 | Antiexpert*.ti,ab. | 0 |
| 64 | (Hostil* adj2 expert*).ti,ab. | 4 |
| 65 | (Trust* adj2 expert*).ti,ab. | 1,133 |
| 66 | Anti-scien*.ti,ab. | 378 |
| 67 | Antiscien*.ti,ab. | 262 |
| 68 | (Hostil* adj2 scien*).ti,ab. | 40 |
| 69 | (Trust* adj2 scient*).ti,ab. | 756 |
| 70 | (Hostil* adj2 research*).ti,ab. | 144 |
| 71 | (Trust* adj2 research*).ti,ab. | 3,659 |
| 72 | Anti-business*.ti,ab. | 24 |
| 73 | Antibusiness*.ti,ab. | 17 |
| 74 | (Hostil* adj2 business).ti,ab. | 23 |
| 75 | (Trust* adj2 business).ti,ab. | 313 |
| 76 | Anti-government.ti,ab. | 174 |
| 77 | Antigovernment.ti,ab. | 63 |
| 78 | (Hostil* adj2 government).ti,ab. | 30 |
| 79 | (Trust* adj2 government).ti,ab. | 2,391 |
| 80 | Culture war*.ti,ab. | 614 |
| 81 | Woke.ti,ab. | 2,866 |
| 82 | Conspiracy.ti,ab. | 5,745 |
| 83 | Mainstream media.ti,ab. | 1,957 |
| 84 | Antifa.ti,ab. | 3 |
| 85 | Antifacist*.ti,ab. | 0 |
| 86 | Social populism.ti,ab. | 0 |
| 87 | Far-left.ti,ab. | 332 |
| 88 | Farleft.ti,ab. | 0 |
| 89 | Anti-corporat*.ti,ab. | 57 |
| 90 | Anticorporat*.ti,ab. | 26 |
| 91 | Anti-left*.ti,ab. | 23 |
| 92 | Antileft*.ti,ab. | 1 |
| 93 | Anti-conservative*.ti,ab. | 189 |
| 94 | Anticonservative*.ti,ab. | 139 |
| 95 | (Hostil* adj2 left*).ti,ab. | 9 |
| 96 | (Hostil* adj2 conservative*).ti,ab. | 10 |
| 97 | or/1-96 [POPULIST ATTITUDES] | 139,662 |
| 98 | Abortion.ti,ab. | 139,846 |
| 99 | Sexual health.ti,ab. | 48,617 |
| 100 | Reproductive health.ti,ab. | 60,012 |
| 101 | Pregnan*.ti,ab. | 1,600,411 |
| 102 | Contracepti*.ti,ab. | 196,376 |
| 103 | Covid*.ti,ab. | 947,013 |
| 104 | Corona*.ti,ab. | 1,623,075 |
| 105 | SARS-CoV-2.ti,ab. | 306,164 |
| 106 | Pandemic.ti,ab. | 604,598 |
| 107 | Influenza.ti,ab. | 306,224 |
| 108 | Flu.ti,ab. | 51,962 |
| 109 | Facemask.ti,ab. | 4,578 |
| 110 | Face-mask.ti,ab. | 11,509 |
| 111 | Isolation.ti,ab. | 783,532 |
| 112 | Social distanc*.ti,ab. | 34,998 |
| 113 | Non-pharmac*.ti,ab. | 53,562 |
| 114 | Nonpharmac*.ti,ab. | 31,146 |
| 115 | Infection control*.ti,ab. | 76,614 |
| 116 | Vaccin*.ti,ab. | 1,134,105 |
| 117 | Anti-Vaccination Movement/ | 843 |
| 118 | Vaccination Refusal/ | 3,624 |
| 119 | Vaccination Hesitancy/ | 7,529 |
| 120 | Immunis*.ti,ab. | 38,577 |
| 121 | Immuniz*.ti,ab. | 391,916 |
| 122 | MMR.ti,ab. | 32,803 |
| 123 | Measles.ti,ab. | 70,542 |
| 124 | Mumps.ti,ab. | 24,999 |
| 125 | Rubella.ti,ab. | 36,467 |
| 126 | Climate change prevention.ti,ab. | 29 |
| 127 | Environmental protection.ti,ab. | 43,669 |
| 128 | Pollution.ti,ab. | 339,909 |
| 129 | Emissions.ti,ab. | 193,137 |
| 130 | 15-minute cit*.ti,ab. | 30 |
| 131 | fifteen-minute cit*.ti,ab. | 2 |
| 132 | road safety.ti,ab. | 10,039 |
| 133 | speed camera*.ti,ab. | 4,144 |
| 134 | enforcement camera*.ti,ab. | 71 |
| 135 | mental health.ti,ab. | 810,626 |
| 136 | mental illness*.ti,ab. | 153,894 |
| 137 | anxiety.ti,ab. | 926,472 |
| 138 | depress*.ti,ab. | 1,804,599 |
| 139 | physical* active*.ti,ab. | 36,559 |
| 140 | physical* exercise*.ti,ab. | 61,989 |
| 141 | diet.ti,ab. | 1,257,289 |
| 142 | nutrition.ti,ab. | 648,759 |
| 143 | obes*.ti,ab. | 1,186,981 |
| 144 | over-weight*.ti,ab. | 2,782 |
| 145 | overweight*.ti,ab. | 300,837 |
| 146 | cigarette*.ti,ab. | 247,693 |
| 147 | tobacco.ti,ab. | 333,812 |
| 148 | vaping.ti,ab. | 9,777 |
| 149 | vape*.ti,ab. | 4,754 |
| 150 | smok*.ti,ab. | 1,031,728 |
| 151 | (Illegal adj2 drug*).ti,ab. | 10,063 |
| 152 | (Illicit adj2 drug*).ti,ab. | 46,177 |
| 153 | (Recreational adj2 drug*).ti,ab. | 9,961 |
| 154 | substance*.ti,ab. | 1,049,837 |
| 155 | alcohol*.ti,ab. | 1,233,195 |
| 156 | Screening*.ti,ab. | 1,933,521 |
| 157 | Gun control.ti,ab. | 1,154 |
| 158 | Firearm ban.ti,ab. | 0 |
| 159 | Gun-violence prevention.ti,ab. | 231 |
| 160 | Gun safety.ti,ab. | 403 |
| 161 | Gambling.ti,ab. | 36,229 |
| 162 | Betting.ti,ab. | 3,786 |
| 163 | Fluoridation.ti,ab. | 7,837 |
| 164 | Fluoridi#ation.ti,ab. | 23 |
| 165 | Health-care access.ti,ab. | 11,361 |
| 166 | Healthcare access.ti,ab. | 9,755 |
| 167 | or/98-166 [PUBLIC HEALTH INTERVENTIONS] | 15,938,673 |
| 168 | Attitude*.ti,ab. | 764,403 |
| 169 | Belief*.ti,ab. | 436,984 |
| 170 | View*.ti,ab. | 1,812,063 |
| 171 | Acceptab*.ti,ab. | 681,917 |
| 172 | Adhere*.ti,ab. | 695,082 |
| 173 | Effect*.ti,ab. | 22,972,732 |
| 174 | Effective*.ti,ab. | 7,229,965 |
| 175 | Outcome*.ti,ab. | 6,981,530 |
| 176 | Impact*.ti,ab. | 5,100,904 |
| 177 | Benefit*.ti,ab. | 2,746,378 |
| 178 | or/168-177 [OUTCOME] | 33,192,990 |
| 179 | 97 and 167 and 178 | 23,208 |
| 180 | limit 179 to yr="2008-Current" | 20,751 |

1. Reference: Campbell M, McKenzie JE, Sowden A, et al. Synthesis without meta-analysis (SWiM) in systematic reviews: Reporting guideline. *The BMJ*. 2020;368. doi:10.1136/bmj.l6890 [↑](#footnote-ref-1)
2. Includes Econlit, Embase, Global Health, Ovid MEDLINE (ALL), PsycINFO, Social Policy and Practice [↑](#footnote-ref-2)
