# Supplementary material for: Exploring how to widen the acceptability of public health interventions: a systematic review protocol
Source: BMJ Open. 2024 Nov 9;14(11):e088418. doi: 10.1136/bmjopen-2024-088418 (PMC11551972; doi:10.1136/bmjopen-2024-088418)
Supplement: Supplementary file 2 [file bmjopen-2024-088418.reviewer_comments.pdf]

PEER REVIEW HISTORY

BMJ Open publishes all reviews undertaken for accepted manuscripts. Reviewers are asked to complete a checklist review form and are provided with free text boxes to elaborate on their assessment. These free text comments are reproduced below.

ARTICLE DETAILS

Title (Provisional)

Exploring how to widen the acceptability of public health interventions: a systematic review protocol

Authors

Conway-Moore, Kaitlin; Graham, Fiona; McKinlay, Alison R; Birch, Jack; Oliver, Emily; Bambra, Clare; Kelly, Michael P; Bonell, Chris

---

VERSION 1 - REVIEW

**Reviewer**	**1**
**Name**	**Ge, Shaoqing**
**Affiliation**	**University of Washington, Biobehavioral Nursing and Health Informatics**
**Date**	**26-Aug-2024**
**COI**	**N/A**

This manuscript is a protocol for a systematic review that aims to explore the acceptability of public health interventions. The protocol is well-developed with a strong and clear background session. The methods are appropriate for addressing the research aims. The strengths and limitations of the study are presented.

In summary, given the significance of the topic and the quality of the manuscript, I would recommend considering publishing this research protocol.

**Reviewer**	**2**
**Name**	**Holst, Jens**
**Affiliation**	**Fulda University of Applied Sciences**
**Date**	**02-Sep-2024**
**COI**	**None**

Thank you for giving me the opportunity to review and comment on the design of a very interesting, innovative and relevant study. The overall idea and design is well thought out

and compelling, with the exception of two major and very few minor issues that need to be reconsidered:

The research question formulated by the authors is very ambitious and likely to not being adequately answered as long as it includes the "effects of public health interventions". The study design and the very nature of the approach will only be able to provide satisfactory results about the linkage of populism to both acceptance and adherence of PH interventions but is unlikely to adequately include any causal assessment of the effects, which of course depend on many other factors. Even in the case of COVID-19, PH interventions lead to quite different effects in different countries or societies; moreover, a serious analysis of the linkage between populism and effects od PH interventions will have to distiguish between the various types of interventions, because they each show different effectiveness. In a nutshell: I would recommend the authors to focus on acceptance and adherence and not include the effectiveness in the research question; the conclusions to be drawn from the study results should rather be referred to in the discussion. It would be interesting and relevant to get to know the findings of the initial scoping review mentioned.

Another challenge to be aware of will be the difficulty of adequately taking into account the strategies applied for the PH interventions; they definitely differ between countries and are likely to have an impact on the acceptance and adherence of interventions. In other words: How to measure the negative effects e.g. of the media overkill many people, regardless of their political conviction, felt during the pandemic. Moreover, the authos will have to be aware of a double publication bias particularly during the COVID-19 crisis because anecdotal observations lead to the assumption that biomedical and traditional epidemiological hardline scholars were more likely and more successful in getting their stuff published.

Some additional suggestions: If OECD countries will be included, ths study will not only comprise high but also upper-middle income countries.

Page 9: Would suggest deleting "the success of" in "... for how populist attitudes affect the success of

public health interventions".

VERSION 1 - AUTHOR RESPONSE

Reviewer 1 Comments

Reviewer Comments	Author Response	Revised Text
This manuscript is a protocol for a systematic review that aims to explore the acceptability of public health interventions. The	Thank you very much. We appreciate your time in reviewing the protocol and providing your feedback.	N/A

protocol is well-developed with a strong and clear background session. The methods are appropriate for addressing the research aims. The strengths and limitations of the study are presented.<br>In summary, given the significance of the topic and the quality of the manuscript, I would recommend considering publishing this research protocol.		

Reviewer 2 Comments

**Reviewer Comments**	**Author Response**	**Revised Text**
The research question formulated by the authors is very ambitious and likely to not being adequately answered as long as it includes the "effects of public health interventions". The study design and the very nature of the approach will only be able to provide satisfactory results about the linkage of populism to both acceptance and adherence of PH interventions but is unlikely to adequately include any causal assessment of the effects, which of course depend on many other factors. Even in the case of COVID-19, PH interventions lead to quite different effects in	Our review aims to synthesise the evidence on how populist attitudes influence the uptake, acceptability, adherence and/or effectiveness of public health interventions. If we find evidence on how populist attitudes affect effectiveness we will review and synthesise it alongside the other aspects. If we do not we will restrict our attention to the other aspects. In relation to effectiveness, the evidence we would be including relates to studies that examine how the effectiveness of public health interventions is moderated by populist	“Narrative synthesis will focus on how the uptake, acceptability, adherence and/or effectiveness of public health interventions appear to be affected by the holding of populist views among potential beneficiaries within each study.”

different countries or societies; moreover, a serious analysis of the linkage between populism and effects od PH interventions will have to distiguish between the various types of interventions, because they each show different effectiveness. In a nutshell: I would recommend the authors to focus on acceptance and adherence and not include the effectiveness in the research question; the conclusions to be drawn from the study results should rather be referred to in the discussion.	attitudes. In other words, we are interested in effect modification or interaction. These analyses would be comparing effects within studies among different subgroups (with and without populist attitudes) rather than comparing across studies. This is perfectly feasible in principle but we shall see whether there are such studies out there to include. We have amended our methods section to make clearer that we will be comparing uptake, acceptability, adherence and/or effectiveness within studies (see **pg. 7, 352-354**).	
It would be interesting and relevant to get to know the findings of the initial scoping review mentioned.	This is not a published review with formal methods or synthesis but rather a preliminary set of searches which we used to establish that there is at least some pertinent evidence out there and to inform what search terms we used. We have amended the language throughout the protocol to make this clearer (see, for example, **pg. 4, lines 171-176**).	"While a set of preliminary searches of published literature conducted by the authors of this protocol, along with media coverage on the topic, has identified interventions focused on vaccination, climate change, sexual and reproductive healthcare, non-pharmaceutical-based infection control, tobacco and alcohol consumption, and diet and exercise as key areas of focus, this study will take a broader approach to include all potentially relevant public health interventions."
Another challenge to be aware of will be the	The reviewer is correct that the uptake,	"As part of this synthesis, we will also reflect on the nature of

difficulty of adequately taking into account the strategies applied for the PH interventions; they definitely differ between countries and are likely to have an impact on the acceptance and adherence of interventions. In other words: How to measure the negative effects e.g. of the media overkill many people, regardless of their political conviction, felt during the pandemic. Moreover, the authos will have to be aware of a double publication bias particularly during the COVID-19 crisis because anecdotal observations lead to the assumption that biomedical and traditional epidemiological hardline scholars were more likely and more successful in getting their stuff published.	acceptability, adherence and/or effectiveness of public health interventions will be affected not only by the attitudes of those they target but also by their design. Our study will be comparing within each included study how the uptake, acceptability, adherence and/or effectiveness of an intervention differs among population subgroups defined in terms of their possession or not of populist attitudes. Hence the intervention characteristics will be constant while the attitudes will vary. This makes for a fair comparison. When we report our findings about how populist attitudes affect the uptake, acceptability, adherence and/or effectiveness of public health interventions, we will reflect on what were the interventions in question. We might find for example that very restrictive infection control measures go down very badly among those with populist attitudes but that less restrictive measures do not. We have amended our methods section to make clearer that when reporting our findings,	the interventions on which the included studies report, as these may be different based on study setting/location.”

	we will reflect on the nature of the interventions on which the included studies report (see **pgs. 7-8, lines 356-363**).	
Some additional suggestions: If OECD countries will be included, ths study will not only comprise high but also upper-middle income countries.	This point is well noted. We have removed the "high income" specification from the text (see **pg.5, line 209**).	"Types of participants<br><br>Given our desire to ultimately identify evidence applicable to a UK policy context, studies will be included if participants are adults living in countries as defined by membership in the Organisation for Economic Co-operation and Development (OECD)."
Page 9: Would suggest deleting "the success of" in "... for how populist attitudes affect the success of public health interventions".	This point is well noted. We have amended the language as suggested (see **pg.8, lines 368-370**).	"The overarching aim of this narrative synthesis will be the development of a conceptual framework for that can be used to inform policy strategies aimed at widening the impact of key public health interventions."
